# Resources and Recommendations for Using Transcriptomics to Address Grand Challenges in Comparative Biology

**DOI:** 10.1093/icb/icw083

**Published:** 2016-09-17

**Authors:** Donald L. Mykles, Karen G. Burnett, David S. Durica, Blake L. Joyce, Fiona M. McCarthy, Carl J. Schmidt, Jonathon H. Stillman

**Affiliations:** *Department of Biology, Colorado State University, Fort Collins, CO 80523, USA; ^†^Grice Marine Laboratory, College of Charleston, Charleston, SC 29412, USA; ^‡^Hollings Marine Laboratory, Charleston, SC 29412, USA; ^§^Department of Biology, University of Oklahoma, Norman, OK 73019, USA; ^¶^BIO5 Institute, School of Plant Sciences, University of Arizona, Tucson, AZ 85721, USA; ^‖^School of Animal and Comparative Biomedical Sciences, University of Arizona, Tucson, AZ 85721, USA; ^**^Department of Animal and Food Sciences, University of Delaware, Newark, DE 19716; ^††^Romberg Tiburon Center for Environmental Studies and Department of Biology, San Francisco State University, Tiburon, CA 94920, USA; ^‡‡^Department of Integrative Biology, University of California Berkeley, Berkeley, CA 94720, USA

## Abstract

High-throughput RNA sequencing (RNA-seq) technology has become an important tool for studying physiological responses of organisms to changes in their environment. *De novo* assembly of RNA-seq data has allowed researchers to create a comprehensive catalog of genes expressed in a tissue and to quantify their expression without a complete genome sequence. The contributions from the “Tapping the Power of Crustacean Transcriptomics to Address Grand Challenges in Comparative Biology” symposium in this issue show the successes and limitations of using RNA-seq in the study of crustaceans. In conjunction with the symposium, the Animal Genome to Phenome Research Coordination Network collated comments from participants at the meeting regarding the challenges encountered when using transcriptomics in their research. Input came from novices and experts ranging from graduate students to principal investigators. Many were unaware of the bioinformatics analysis resources currently available on the CyVerse platform. Our analysis of community responses led to three recommendations for advancing the field: (1) integration of genomic and RNA-seq sequence assemblies for crustacean gene annotation and comparative expression; (2) development of methodologies for the functional analysis of genes; and (3) information and training exchange among laboratories for transmission of best practices. The field lacks the methods for manipulating tissue-specific gene expression. The decapod crustacean research community should consider the cherry shrimp, *Neocaridina denticulata*, as a decapod model for the application of transgenic tools for functional genomics. This would require a multi-investigator effort.

## Introduction

The Animal Genome to Phenome (AG2P) Research Coordination Network (RCN) was created to foster greater communication and collaboration among investigators faced with the challenge of linking “-omics” (genomics, transcriptomics, and proteomics) data to whole-organism phenotype and performance. The effort spans both model and non-model organisms with the intent that the experience obtained studying model organisms can hasten discovery in non-model organisms. Here, “non-model” is defined as those organisms for which a genome sequence is lacking or incomplete, and for which we lack efficient methods for altering the expression of a specific gene in a specific tissue at a specific time to test gene function. The AG2P RCN provides organizational support for collaboration and networking among biologists by way of workshops and meetings. The RCN is intent on fostering the development of mechanistic approaches and infrastructure for understanding of how changes at the genomic level are linked to physiological processes in non-model organisms.

The AG2P RCN held its first workshop at the January 2016 meeting of the Society for Integrative and Comparative Biology in Portland, OR. The objective was to reach out to and query Society for Integrative and Comparative Biology (SICB) members attending the all-society symposium, “Tapping the Power of Crustacean Transcriptomics to Address Grand Challenges in Comparative Biology,” organized by four members of the AG2P RCN executive committee (Burnett, Durica, Mykles, and Stillman). Attendees at the workshop identified some of the impediments that currently limit the use of transcriptomics by integrative organismal biologists. The participants ranged from graduate students to senior PIs, with experience using RNA-sequencing (RNA-seq) technology ranging from none to many years. Here, we summarize the input received, describe the resources available on the CyVerse platform, and make recommendations for future directions that will accelerate an understanding of genome to phenome relationships in decapod crustaceans.

## Input from the integrative organismal biology community

The AG2P RCN group organized an informal lunch, a feedback booth in the exhibit hall, and workshop at the 2016 SICB annual meeting in Portland to gather comments from organismal biologists on their experiences using high-throughput next generation sequencing (NGS) technologies in their research. The speakers in the crustacean transcriptomics symposium participated in the discussions. Many of the attendees study non-model organisms, in which there are no, or only partial, genome sequences available. *De novo* assembly of RNA-seq data has enabled researchers to catalog and quantify transcripts without relying on the genome, but the approach does have some drawbacks. Particular challenges include inferring gene homology across species, analysis of regulatory RNA (e.g., siRNA), and assessing alternative splicing within species.

**Fig. 1.**
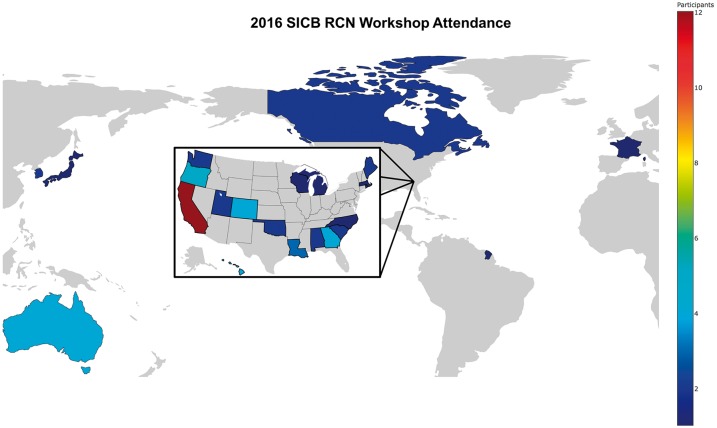
Geographical distribution of the participants of the 2016 SICB Tapping the Power of Crustacean Transcriptomics workshop organized by the Animal Genome to Phenome Research Coordination Network.

Discussion participants studied a great variety of organisms, including crustaceans, insects, corals, sponges, mammals, and fish, and represented different geographical regions (Fig. 1). They fell into two broad categories of users: (1) newcomers who see the potential of the technology for their research questions, but do not know where to start, and (2) experienced users who are frustrated by the limitations of the current tools and hoping for improvements. It was clear that the experience level correlated to what the user wants from bioinformatics tools; the novice has no interest, at least at first, in becoming a bioinformatician and prefers a standardized pipeline with default parameters, whereas the experienced user is not averse to writing code and prefers a pipeline with options and flexibility. It is unrealistic to expect that a standardized pipeline would be broadly applicable without some capability for customization by the user. Seven needs were identified from the comments and discussions.

### Knowing where to start

There is a need to lower the threshold for new users who want to start using high-throughput sequencing technologies. They need guidance on library construction, sequence depth, library size, contamination, quality control, and the number of replicates. New users want a “beginners’ bioinformatics pipeline” with common workflows and tools for validation and post-analysis of assemblies, collection of metadata for RNA-seq datasets (e.g., Minimum Information About a Microarray Experiment, or MIAME-like, protocols (Brazma et al. 2001), decontamination and filtering of datasets, and ways for use and sharing of data.

### Assessing the quality of outputs

There is a need for best practices and experimental design guidelines for evaluating the quality of RNA-seq data and assemblies. For a novice, it is a challenge to know what level of quality is acceptable, in particular because the answer may depend on the questions one is asking of the RNA-seq data. Tools optimized for use in non-model species are needed to assess data or benchmark accuracy of outcomes. What cutoffs (e.g., *P* values) should be used? There should be well-defined criteria for including transcripts that are novel and/or expressed at low levels.

### Structural annotation

Transcript sequences are often annotated before a functional analysis is done, which requires comparative and phylogenetic analyses using nucleotide and protein databases. Users want to know the best practices for the identification of orthologs and homologs across species. Are there standard procedures and criteria for assigning transcripts to a particular gene? What is the optimal balance between reducing transcript redundancy, while not eliminating alternatively-spliced isoforms? What is the best practice for assembling a “reference” transcriptome? Should one combine RNA-seq data from different tissues, different conditions, and data sets from different labs?

### Functional analysis

Another need articulated by many at the meeting was how to get from gene lists back to the biology—establishing functional annotation of transcripts relevant to the biological pathways being studied in non-model organisms. Sorting through the thousands of transcripts identified in a typical RNA-seq data set to identify a small subset of genes that regulate a physiological process or response to an environmental signal is time consuming and is limited by the tools that are available. Workflows and guidelines are needed and statistical tools are needed to identify gene interactions and dynamic changes in gene networks.

### Knowing when an analysis is finished

New software packages and software updates are constantly being introduced. Oftentimes, a new introduction requires reanalysis of the data. Guidelines are needed for determining when an analysis is good enough to start testing predictions. Once the analysis is finalized, users want to know what should be shared and when. The truth is that analysis of RNA-seq data is never really done. Authors should make their data sets available for reanalysis. CyVerse can provide a secure means to manage and share data with collaborators. The user controls the permission process allowing access to the data. Although CyVerse is not designed to be a long-term repository, tools are being deployed at CyVerse to facilitate movement of assemblies and large data sets from the Data Store to long-term repositories such as the National Center for Biotechnology Information (NCBI).

### The challenges of bioinformatics

There is confusion about which bioinformatics tools to use. Different software packages that do the same thing (e.g., sequence annotation and differential expression) often yield different sets of transcripts. Should one use only the subset of transcripts identified in common among different software packages? One must deal with many different file formats and know which ones to use. How does one cluster transcripts, and which sequences should serve as the “reference”? Which variants are biologically meaningful and which are computational artifacts?

### Training

There is a need for workshops and online videos/webcasts with step-by-step training in RNA-seq data analysis, including statistics. We need to promote the use of networking tools, such as Trellis, a digital platform being developed by the American Association for the Advancement of Science (https://www.trelliscience.com/#/site-home), to facilitate collaborations and consultations with experts.

## Resources available to the research community on CyVerse (formerly iPlant)

**Fig. 2.**
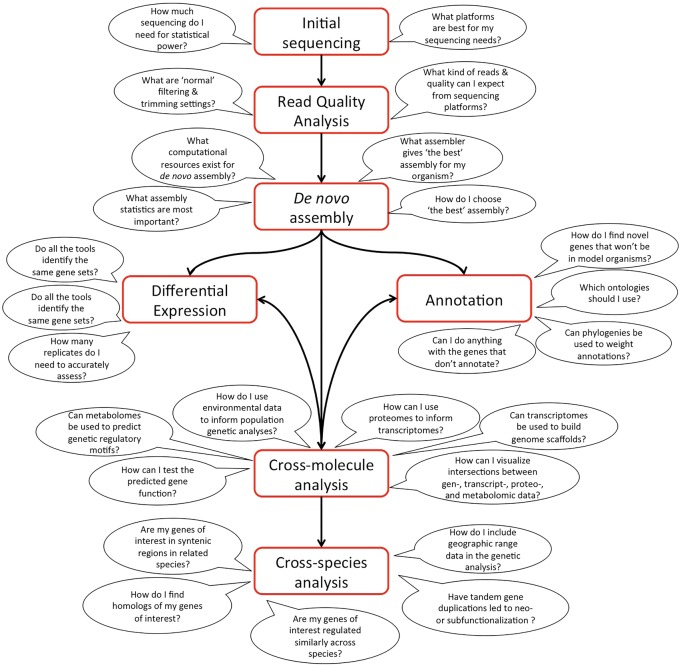
Questions associated with each step of bioinformatics analysis of RNA-seq data. Comments from 2016 SICB meeting participants were collected and categorized by the Animal Genome to Phenome Research Coordination Network. The discussions at the workshop and comments received during the meeting are arranged as questions associated with steps in the RNA-seq assembly and analysis pipeline.

It was clear from the comments received that many researchers were not aware of the resources currently available for bioinformatic analysis and training for those studying the transcriptomes of non-model organisms. If the resources were known, a common complaint was that researchers had difficulty in evaluating and choosing the best tools to use for their particular application. Both of these problems are attributed to the lack of a single comprehensive platform with a user-friendly interface. CyVerse (formerly iPlant) aspires to be that “one-stop shop” for bioinformatics tools, analogous to the role the NCBI GenBank serves for archiving and analyzing nucleotide sequences. CyVerse designs and deploys cyberinfrastructure in order to deliver computational resources for empowering scientific research. The goal of CyVerse is to democratize biocomputing across the life sciences by providing accessible computing and data management resources for all biologists. CyVerse is currently working with the animal genomics community to identify their computational and bioinformatic needs. The needs identified during the 2016 SICB conference were primarily focused on computational pipelines for analyzing genetic data derived from under-served organisms (Fig. 2).

### Sequencing and assessing read quality

There are myriad sequencing platforms and library types that can be sequenced (e.g., RNA-seq, bisulphite sequencing, and ChIP-seq). One need identified at SICB is tutorials that explain sequencing platform outputs, which platform(s) and technologies are needed for robust assembly of sequencing data derived from non-model organisms, and whether the data provided will allow for enough statistical power to detect differences between experimental treatments. CyVerse has sequence analysis programs such as FASTQC, which allow researchers to evaluate sequence quality (Andrews 2010; http://www.bioinformatics.babraham.ac.uk/projects/fastqc/). High-quality sequence data is crucial in assembling genomes or transcriptomes in organisms that do not have reference sequences.

### De novo assembly

*De novo* genome and transcriptome assemblers match high-quality reads to every other read in a pool to build larger contiguous sequences. SICB researchers expressed the need for tutorials that explain the differences between assemblers, and how these differences might affect an assembly. There was also a need to have empirical evidence to decide when genomes and transcriptomes are “complete”. While these issues will require further investigation, especially considering that different organisms will have different requirements based on the size and complexity of their genomes, CyVerse offers several *de novo* assemblers and computational resources to accomplish assemblies in a relatively short time. This will allow researchers to iterate across different assemblers and assembly conditions, enabling experimentation to determine the best assembly for each study.

### Workflows for functional and comparative analysis of transcriptome data

Ultimately, assembled genomes and transcriptomes need to be turned into biological concepts and insight. Many researchers are interested in differential expression of genes between tissues, individuals, treatments, or populations. CyVerse offers several differential expression analysis packages, e.g., DESeq or EdgeR. Functional annotation attempts to describe the biological function of identified genes. CyVerse supports popular annotation tools such as InterProScan (Hunter et al. 2009) and Trinotate (Grabherr et al. 2011; Haas et al. 2013). In addition, there are also other platforms serving animal research communities that conduct functional annotation. Currently, CyVerse is partnering with AgBase, a curated, web-based resource that specializes in functional annotation of animal genomes (McCarthy et al. 2010). Together CyVerse and AgBase are developing functional annotation and functional analysis workflows. Lastly, comparing *de novo* genomes and transcriptomes to model organisms or community-specific genomes was of interest to SICB researchers. The Comparative Genomics (CoGe) platform can access data from the CyVerse Data Store, and has a suite of tools to manage, compare, and visualize genomes (Lyons and Freeling 2008). Additionally, CoGe has several workflows that will align RNAseq data to genomes, calculate expression profiles, identify SNPs and methylation sites, and compute basic population genetics summary statistics.

### Integration of “-omics” data types

SICB researchers identified the importance of integrating data from genomic, transcriptomic, proteomic, metabolomic, and even geographic locale data sets. These “cross-molecule” analyses allow for analyzing complex phenotypes, understanding animal distribution and population genetics, and developing integrated models that span previously isolated disciplines of biology. Accomplishing these types of complex analyses requires fostering interoperability among data integration, visualization, and analysis tools being developed by community members and existing platforms that deliver cyber-infrastructure. Creating an ecosystem of interoperability will require learning best practices for tool development, application programming interfaces (APIs), and data management. CyVerse fosters creating interoperability among bioinformatics resources through its Powered by CyVerse program (http://www.cyverse.org/powered-by-cyverse). Researchers can interface with the CyVerse cyberinfrastructure in several ways (http://www.cyverse.org/collaborate). CyVerse APIs provide direct programmatic access to the CyVerse cyberinfrastructure to connect platforms or build new community-specific platforms (http://www.cyverse.org/science-apis). Researchers can also submit proposals for direct collaboration to address computational needs of a scientific project (http://www.cyverse.org/content/get-extended-collaborative-support).

### Community support

Lastly, documentation and instructional material for using bioinformatics tools were also of interest to SICB researchers. Specifically, the need for peer-reviewed, impartial protocol manuscripts was voiced from nearly all community members. Many of the tools in CyVerse have associated documentation that describes the tool and its use. Training videos, tutorials, and past workshops are also available on the CyVerse wiki (http://www.cyverse.org/learning-center/get-help). CyVerse also has a forum where users can post technical or scientific questions (http://ask.cyverse.org/questions/). CyVerse’s Scientific Analysts monitor this forum and respond to questions that are posted. Researchers can also request CyVerse workshops to familiarize themselves with tools available (http://www.cyverse.org/connect). In addition, CyVerse’s education platform, DNA Subway, provides a simplified graphical user interface for training in bioinformatics workflows (http://www.cyverse.org/dna-subway).

## Future directions and recommendations

Transcriptomics using NGS technology has revealed genes involved in stress response, reproduction, development, molting and growth, limb regeneration, immune response, endocrinology, and nutrition and digestion in decapod crustaceans (Sagi et al. 2013; Tom et al. 2013; Durica et al. 2014; Ghaffari et al. 2014; Hao et al. 2014; Lv et al., 2014, 2015; Shen et al. 2014; Song et al. 2014; Tom et al. 2014; Wei et al. 2014a, 2014b; Abehsera et al. 2015; Chandler et al. 2015; Christiaens et al. 2015; Huang et al. 2015; Johnson et al. 2015; Li et al. 2015a, 2015b; Ventura et al. 2014, 2015; Verbruggen et al. 2015; Wang et al. 2015; Das et al. 2016a). It is clear from the input received at the SICB meeting and the ten symposium papers in this issue (Armstrong and Stillman 2016; Chandler et al. 2016; Clark and Greenwood 2016; Das and Mykles 2016; Das et al. 2016b; Havird and Santos 2016a, 2016b; Johnson et al. 2016; Powell et al. 2016; Tarrant et al. 2016) that a greater understanding of the relationship between transcriptomic and phenotypic change in decapod crustaceans will not be achieved until more powerful and applicable “-omic” tools and resources are developed. A decapod model in which functional genomics data are used to test physiological effects is necessary for linking genotype to phenotype. While alternative species have been considered, such as the red swamp crayfish *Procambarus clarkii* and the redclaw crayfish, *Cherax quadricarinatus*, Mykles and Hui (Mykles and Hui 2015) argue that the cherry shrimp, *Neocaridina denticulata*, possesses the best combination of traits to facilitate the development of functional genomic tools in decapod crustaceans (see below). We propose three major initiatives.

### Integration of genomic and RNA-seq sequence assemblies for crustacean gene annotation and comparative expression

Functional annotation requires complete genome and transcriptome sequences, which is particularly important for identification of alternatively spliced isoforms and microRNAs. A draft genome for *Eriocheir sinensis*, the Chinese mitten crab, was recently released (Song et al. 2016), but this assembly represents about two-thirds of the estimated genome size and consists of over 17,500 scaffolds with an N50 length of 0.224 Mb. A preliminary genome of *N. denticulata* is available (Kenny et al. 2014), and genes in the juvenoid and ecdysteroid hormone synthetic pathways were identified in the genome (Sin et al. 2015). However, scaffold lengths are too few in number (∼200 with lengths exceeding 5 kb) and too short (e.g., N50 of 0.0004 Mb and a maximum contig length of 0.124 Mb) for genome-scale analysis. Moreover, a complete *N. denticulata* transcriptome is lacking, as are patterns of differential gene expression under a range of conditions. A goal is to obtain a comprehensive catalog of all the genes expressed in the shrimp at all stages of development. Those transcript sequences will also assist in the assembly of genome scaffolds (Xue et al. 2013). A complete genome assembly for the cherry shrimp and other decapods will provide information on gene organization and transcription unit structure that can assist RNA-seq contig assembly. These databases will be useful for investigations on epigenetic changes associated with phenotypic change (eg. ChIP-seq; Pepke et al. 2009).

The integration of genomic and transcriptomic sequences will allow for gene identification, the development of a decapod crustacean orthologous gene database, and functional annotation into gene pathways and networks. Gene ontology (GO) and pathway analysis require manual curation and modification for pathways unique to decapods or arthropods in general, such as neuropeptide signaling and ecdysteroid and juvenoid hormone synthetic pathways (Mykles 2011; Covi et al. 2012; Tom et al. 2013; Sin et al. 2015;Das et al. 2016a). Preliminary comparative transcriptomic studies suggest numerous unannotated, novel genes. Statistical tools must be developed for determining significant changes in gene expression and interactions between genes and networks.

### Development of methodologies for the functional analysis of genes

Standardized methods must be developed to experimentally evaluate gene function. Currently, the only method that has been tested in decapod crustaceans to manipulate gene expression is RNAi (De Santis et al. 2011; Ventura et al. 2011; Pamuru et al. 2012; Das and Durica 2013). The dsRNA must be delivered by injection. A major disadvantage is that one cannot control for systemic effects, as the dsRNA cannot be targeted to a specific tissue. Moreover, there are no methods for over-expression of a gene. The goal is to develop *N. denticulata* into a tractable genetic model through transgenics and mutation screens. Methods used successfully in insects and amphipod crustaceans, such as CRISPR/Cas9, TALEN, and RNAi with tissue-specific expression constructs, may be applied to other crustaceans species (Gaj et al. 2013; Daimon et al. 2015; Enya et al. 2015; Li et al. 2015c; Martin et al. 2016; Valzania et al. 2016). *Neocaridina* *denticulata* has attributes that are essential for transgenic experiments: (1) they are freshwater animals that live in a wide range of temperatures and water chemistries; (2) relatively short intergenerational time (4–5 months); (3) small size for housing thousands of individuals in limited space; (4) fertilized eggs are relatively large and are brooded externally, facilitating the harvest of eggs for microinjection of gene constructs; (5) direct embryonic development; and (6) the cuticle is thin and transparent, which allows for observation of molt stages, gonad development, and tissue expression of reporter genes, such as red fluorescent protein, in live animals. The methods developed in *N. denticulata* have the potential to translate to work on embryos or adults of other, non-model, decapod crustaceans.

### Information and training exchange among decapod crustacean laboratories

Technical procedures developed under initiatives 1 and 2 above must be disseminated through short courses and the CyVerse website. We envision meetings organized to bring together the crustacean biology and evolutionary/developmental biology communities to determine best practices for testing gene function. Workshops, one focusing on bioinformatics (e.g., gene homology and genomic and transcriptomic data), and one focusing on methods (e.g., animal husbandry, embryo manipulation, gene construct delivery, and reporter assays) should be held annually in conjunction with other conferences (e.g., SICB, Plant and Animal Genome Conference, American Physiological Society, The Crustacean Society, and World Aquaculture Society). A wiki hosted by CyVerse is needed to facilitate inter-laboratory communication and share information. The workshops should include graduate students and postdoctoral fellows for acquiring new skills, accessing information and technologies prior to publication, and promoting networking and collaborative opportunities important to career development.

## Conclusions

The genomics resources developed for decapods will have broad application to other non-model organisms. Establishing standard operating procedures for transgenics in *N. denticulata* would have a transformative effect on crustacean research, both basic and applied, with potential ecological and economic benefits. For example, it may be possible to create a rapidly growing shrimp strain for increased aquaculture production by knocking down molt-inhibiting hormone in the eyestalk ganglia. Crustaceans are of extreme economic importance; in 2014 US shrimp imports were valued at $6.7 billion, 2015 Maine landings of American lobster were valued at $500M, and red king crab is the 2nd most valuable marine resource in Alaska after salmon. Development of transgenic methodologies will also be valuable in understanding impacts of environmental factors on natural crustacean populations, their numbers, and their distribution and resilience to change by adaptation or acclimation. For example, overexpression of heat shock proteins can increase tolerance of temperature stress and reduce susceptibility to infection. In their roles both as predators and prey, crustaceans are vital components of the food web. Thus, developing transgenic resources for crustaceans would dramatically advance our basic understanding of the basic biology of non-model organisms, as well as assist in conservation and harnessing these natural resources for the future.
